# Post-mortem to ante-mortem facial image comparison for deceased migrant identification

**DOI:** 10.1007/s00414-024-03286-0

**Published:** 2024-08-16

**Authors:** Caroline Wilkinson, Martina Pizzolato, Danilo De Angelis, Debora Mazzarelli, Annalisa D’Apuzzo, Jessica Ching Liu, Pasquale Poppa, Cristina Cattaneo

**Affiliations:** 1 Face Lab, Liverpool John Moores University, Liverpool, UK; 2 LABANOF, University of Milan, Milan, Italy

**Keywords:** Facial image comparison, Migrant, Disaster victim identification

## Abstract

The identification of deceased migrants is a global challenge that is exacerbated by migration distance, post-mortem conditions, access to ante-mortem data for comparison, inconsistent international procedures and lack of communication between arrival and origin countries. Due to low technology requirements, fast speed analysis and ease of transferring digital data, facial image comparison is particularly beneficial in those contexts, especially in challenging scenarios when this may be the only initial ante-mortem data available to identify the deceased. The Facial Identification Scientific Working Group (FISWG) professional guidelines for facial image comparison were developed for living facial appearance, and, therefore, a tailored protocol for the application of post-mortem to ante-mortem facial image comparison was proposed and evaluated in this research. The protocol was investigated via an inter-observer and an accuracy study, using 29 forensic cases (2001–2020) from the University of Milan, provided by the Laboratory of Forensic Anthropology and Odontology. In order to replicate a migrant identification scenario, each post-mortem subject was compared to all 29 ante-mortem targets (841 comparisons). The protocol guided the practitioner through stages of facial image comparison, from broad (phase 1) to more detailed (phase 3), eventually leading to a decision of ‘exclusion’ or ‘potential match’ for each post-mortem to ante-mortem case (phase 4). In phase 4, a support scale was also utilised to indicate the level of confidence in a potential match. Each post-mortem subject could be recorded with multiple potential matches. The protocol proved to be useful guide for facial image comparison, especially for less experienced practitioners and the inter-observer study suggested good reproducibility. The majority (82–96%) of ante-mortem subjects were excluded at the first stage of the protocol, and 71 full post-mortem to ante-mortem facial image comparisons were carried out. On average, two or three potential matches were recorded for each post-mortem subject. The overall accuracy rate was 85%, with the majority (79%) of ante-mortem non-targets correctly excluded from the identification process. An increased number and quality of available ante-mortem images produced more successful matches with higher levels of support. All potential matches involving non-targets received low levels of support, and for 73% of the post-mortem subjects, the ante-mortem target was the only recorded potential match. However, two ante-mortem targets were incorrectly excluded (one at the first stage of the protocol) and therefore changes to the protocol were implemented to mitigate these errors. A full protocol and a practical recording chart for practitioner use is included with this paper.

**Supplementary Information:**

The online version contains supplementary material available at 10.1007/s00414-024-03286-0.

## Introduction

Economic migration is a critical issue for many European countries and disasters involving migrants present a related global challenge [1]. Migrant disasters can be defined as the multiple deaths of migrants caused by catastrophic circumstances whilst crossing bodies of water or land between origin and destination. These include capsized boats, wildfires, overcrowding (leading to suffocation) and extreme weather conditions. A disaster may include mass casualties in a single incident (e.g., a capsized boat) or multiple deaths over time with the same pattern (e.g., exposure on migrant land routes). Therefore, where migrants travel great distances across and between continents, a large percentage reach the end of their lives, and, whilst sometimes their bodies are found, unfortunately many are lost at sea, in deserts or in wasteland. As a result, many families may never learn of the fate of their relatives, and few migrants who perish are ever positively identified.

In recent years, due to war and socioeconomic crises, there has been an increased use of dangerous maritime routes to Europe across the Mediterranean and via the Canary Islands and hostile land routes to Europe through Turkey, and a related increase in the number of unidentified deceased migrants [2]. However, the number of fatalities that involve migrants fleeing from countries with volatile political and socioeconomic situations is difficult to calculate [3]. The International Organization for Migration (IOM) has estimated that more than 60,000 migrants have died on these journeys with the majority of disasters taking place in the Southern European borders [4], where Italy, Spain, Malta and Greece are the most affected countries [5–7]. On a smaller scale, there has been a significant increase in migrant deaths along the borders between Mexico and the United States in the last decade [4].

In order to address these challenges, the European Cooperation in Science and Technology (COST) Action CA22106 [8] focusing on Migrant Disaster Victim Identification (MDVI), signifies a concerted European effort to enhance inter-agency communication along with new developments in scientific and technical capabilities in forensic science and humanitarian work. COST aims to develop new, efficient methods for identifying deceased migrants by facilitating cooperation and knowledge exchange among researchers, NGOs, forensic experts, and other stakeholders across Europe, thereby addressing this pressing humanitarian issue more effectively. This initiative not only seeks to improve identification processes, but also by ensuring that families can learn the fate of their loved ones, support human dignity and uphold the human rights which are at the forefront of European values.

**Fig. 1:**
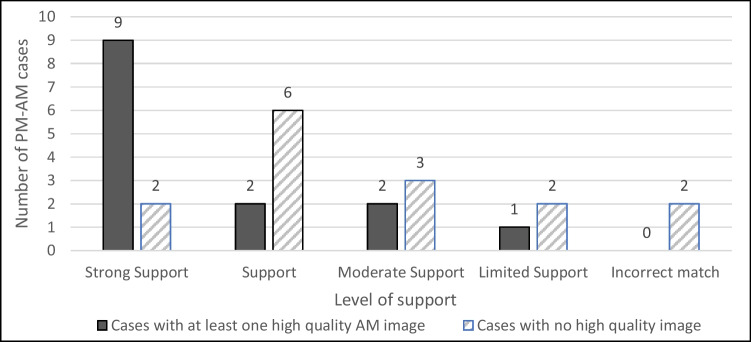
Distribution of post-mortem-ante-mortem (PM-AM) cases with different AM image quality and related level of support for a match

The IOM states that migrants’ rights should be protected during the whole circle of migration [9], and this is especially challenging where the individuals originate from low-income countries, where traditional so-called primary identifiers are limited or absent, and migration is undocumented or unmonitored. The identification of migrants has been defined by identification experts [10] as an “enormous paradox”. It is recognised by the Geneva Convention that identifying a deceased person is a fundamental right and a moral obligation that should be respected without discrimination [11]. However, while identifying the deceased in a mass disaster context is standardised, regulated by internationally accepted procedures and conducted by adequately trained personnel, the identification process becomes slow and lacks urgency in the aftermath of disasters involving migrants [12, 10]. This has led to an incredibly large number of unidentified victims who are buried without a name, leaving families to deal with the psychological consequences of not knowing the fate of their missing loved ones [3]. From a humanitarian and legal point of view, the lack of identification for these victims can be considered a violation of human rights that does not only affect the dignity of the deceased but largely affects the rights of the living [10, 13, 14] (Fig. 1).

Migrant mass disasters include some unique challenges that are different from many ‘usual’ DVI events, although there are similarities with other humanitarian identification challenges, such as the disappeared in countries such as Guatemala, Timor-Leste and Argentina. In these humanitarian identification cases, primary methods of identification are often inappropriate or even impossible, due to legal obstacles and socioeconomic conditions of the countries of origin, fear of reprisals or financial consequences for the migrants’ families and the resulting lack of regular dental treatment, fingerprint resources and medical records. In addition, water-damage, rapid decomposition and delayed access to the migrant bodies may further confound any comparative methods [15]. The IOM states that the majority of deceased migrants are identified visually by family members [16], despite international recognition of significant problems associated with visual recognition of the dead, due to psychological/social pressures and post-mortem (PM) changes [17]. Recently, the division between primary and secondary identifiers has been under discussion in international policymaking groups, such as INTERPOL DVI Working Group.^1^ In a statement published by Forensic Anthropology Society of Europe [18], members argued that the preferred primary identifier cannot always be used to support identification and often secondary identifiers provide sufficient evidentiary value for a conclusive identification with the use of a reliable methodology [18]. There are also significant challenges in relation to the collection of ante-mortem (AM) data from families of the missing in the countries of origin. Since many migrants attempt crossings via trafficking gangs, communication with relatives of the missing may create danger for the family, and relatives may be reluctant to speak to the authorities due to fears of criminal investigation or social exclusion [7].

Craniofacial analysis offers valuable tools for forensic investigation [19, 20], but these techniques have not been widely utilised in MDVI, due to the INTERPOL DVI recommendation for the utilisation of primary identifiers. The contemporary rise of global networks and the use of mobile phone images has led to public acceptance of social media use in the search for missing people; platforms, such as Facebook and Instagram, are frequently utilised to publish AM images of missing people [21]. This has created an opportunity for digital facial analysis to be employed to match PM images to AM images. In the aftermath of a disaster, critical evidence that migrants carry with them, including mobile phones, is often neglected. Much of the useful identification data stored on mobile phones can be accessed in social media or cloud storage, but this data is hardly ever collected, as it would likely involve interviews with families in the country of origin to confirm social media ownership [16] and require permission to access image data. Some European forensic databases have utilised 3D imaging for craniofacial analysis, but these tend to be limited to national forensic casework rather than large-scale disaster scenarios [22, 23].

Facial identification is described as a meticulous, systematic and well-documented manual approach [24, 25] and generally involves comparison between the face of an individual visible in photographs or video frames and facial image/s of the suspected identity [26]. It has become particularly relevant in many courtrooms across the globe due to the increased use and availability of Close Circuit Television (CCTV) footage worldwide [27]. Morphological comparison involves comparing facial images, to assess the similarities and differences by examining the correspondence between shape, appearance, and location of features, as well as identifying features like scars and moles [25, 28, 29]. Facial image comparison experts working for law enforcement, government organizations, and academic institutions and are routinely utilised in legal scenarios. These experts are usually trained in anatomy, principles of image comparison and recognition, and court duties [30]. Their role involves providing expert opinion on the identity of an individual or their potential association with a criminal event by comparing facial images collected at the scene of crime (CCTV or security footage) with those of suspects [31].

Facial image comparison is based on the assumption that the combination of morphological traits observable on faces is unique [25]. However, subjectivity is inherent [32] as the face and its features and shapes are difficult to assess quantitatively [24] due to expression, ageing, facial modifications, image qualities and distractions. Therefore, the final decision is based on the description of similarities and differences and includes a scale of support to express the level of confidence in a match or exclusion. The international joint work of the Facial Identification Scientific Working Group (FISWG) has produced guidelines and protocols for facial comparison [33], including a comprehensive list of facial features to analyse, image quality factors to consider and recommendations for training and competency for experts [34, 35]. The FISWG morphological features list is currently considered the most comprehensive and relevant protocol for the forensic facial comparison [36]. Tests have been carried out to validate the accuracy of the morphological comparison using FISWG guidelines on living individuals; a real-life scenario was simulated with various image types and results suggest an accuracy of 71–99%, with image quality having the greatest impact on accuracy [31]. Currently, there is no minimum requirement for image quality and the images are usually assessed for suitability by the practitioner against factors such as pixel resolution, distortion, pose, lighting and obstruction caused by objects such as hats and sunglasses [37]. The relationship between lower match accuracy and low quality of the image has been confirmed [31, 38–41], and higher accuracy is produced by the combination of morphological analysis and optimal image quality [26, 31]. The accuracy of facial image comparison is considered dependent upon the experience, knowledge and ability of the practitioner [42].

In recent years, there has been a growing interest in using morphological comparison for PM identification [43] particularly for the increasing number of unidentified bodies associated with migrant disasters and other mass fatalities [44]. Images are often a crucial piece of personal data that is easily available to investigators; PM images and descriptions of external morphological features of both the body and face are well-documented by pathologists and first responders [10, 45]. Also, the availability of personal images has significantly increased with the widespread use of social media platforms and smartphones equipped with cameras, meaning that often there is easy access to high-quality images of the faces of missing people. In the context of migration, researchers [44] have highlighted the use of images provided by families to aid in identification and recent MDVI experts in Italy have reported that facial image comparison, particularly where moles and scars were visible, played a fundamental role in supporting DNA analysis for achieving identification [12]. Generally, these elements are listed as comparable facial features that should be analysed during identification of living individuals, but they appear to assume a more significant role in the identification of deceased individuals.

However, the application of facial identification methods to deceased individuals has not received the same consideration as for identification of the living, and this may be due to a lack of knowledge relating to facial decomposition patterns [46]. Decomposition is a natural process that affects an individual after death and gradually leads to the destruction of the tissues [47] following a 5-stage process: fresh, bloat, active decay, advanced decay, and skeletonization [48]. However, these stages are heavily influenced by many intrinsic and extrinsic factors. Intrinsic factors include weight and size of the body, posture of the body at death, and clothing. Extrinsic factors include the environment, temperature, weather, humidity, type of burial, level of insect, and other animal activity [49–51]. PM changes are generally divided into early, mid and late changes. Early PM changes usually appear within 2 h from death, and are characterised by biomolecular changes, visible skin pallor due to cessation in blood circulation, as well as the purging of stomach material and general flaccidity [47]. Mid PM changes include algor mortis, rigor mortis and livor mortis, and later PM changes include putrefaction, mummification and skeletonisation. Only two studies have attempted to quantify the degree of early decomposition changes that affect the face: one in the field [52] and one in a mortuary setting [46]. These studies used 3D laser scans to evaluate patterns of facial decomposition, and, despite the different environments and timelines, both determined the major changes as bloating at the lateral parts of the face, swelling at the orbits with darkening of the eyeballs, and shrinkage at the midline region of the face and upper lips.

The term “skin mark” encompasses a variety of skin irregularities such as moles (or nevi), scars, discolouration, acne, eczema lentigines, cherry hemangiomas, and seborrheic keratoses, with some of them being less stable through time [53]. Skin marks such as moles, blemishes and scars can be powerful morphological elements that provide an important support for facial identification [54, 55]. Assessing the nature of skin marks from a photograph, especially if photographs are of low quality, is difficult, and experts suggest using only recent photographs for skin mark comparison. Researchers [56] have examined the significance of position, shape, and incidence of scars and nevi on the dorsum of the hand and suggest that these characteristics can be crucial in identification. Scars and tattoos were accepted as the sole means of identification in 2% of the victims of the aeroplane disaster in Linate, Milan, 2001 [57] and the early stages of identification in the terroristic attacks in Paris between 2015 and 2016 were enhanced by using strong secondary identifiers, such as tattoos and scars [58]. A similar approach has been utilised in challenging migrant identifications in Greece, where the initial recognition of personal belongings through photographs provided by family members, served as indicative identification later confirmed by genetic kinship analysis [6]. Reviews of DVI procedures [59] in low-resource countries, emphasise the importance of strong secondary identifiers, particularly in situations where primary methods cannot be used. These facial marks are usually recorded during the PM analysis of the victims; however, their stability can be influenced by the decomposition process [17]. Facial marks, such as moles and scars and any blemish visible on the skin, are among the most common features across different populations and these are often visible in photographs [56, 60, 61]. In the same way, facial lines or creases have been shown to be consistent enough to be used for human identification [62] even with early decomposition effects, such as bloating.

Another element of the face that has been found particularly unique is the human ear. The stability of this feature throughout a lifetime, as well as the stability maintained with different facial expressions [63, 64] make the ear a useful biometric. The individuality of the ears has been observed in different populations [65–69] and between monozygotic twins [70], but also for the same individual, where right and left ears were not identical [66, 71]. The morphology of the external ear has been analysed in forensic cases to aid identification, and this has been accepted in the courtroom as identification evidence [72] in cases of robbery (2D-2D image comparison), severe mutilations [67] and fire damage [73]. 3D-2D comparisons have been shown to be more reliable than 2D-2D image comparisons [74] in cases utilising ears for identification.

Facial image comparison is particularly beneficial in those contexts where there is a substantial lack of AM data, as is often the case of migration victims [57]. It is in these cases, experts state that images are one of the most abundant, and in some cases, the only data available to identify the victims [12]. The application and value of facial image comparison to the identification of the deceased have not been investigated formally. The available FISWG guidelines are developed on living facial appearance and therefore a tailored protocol for the application of PM-AM facial image comparison is proposed and evaluated in this research.

This study aimed to evaluate the reliability and reproducibility of a designed facial image comparison protocol for the identification of unidentified bodies with early post-mortem changes.

## Materials and method

A protocol was developed for PM-AM facial image comparison utilising an Excel document with drop-down boxes and a guidance document with reference images. The protocol was evaluated via an inter-observer study alongside an identification study, using AM and PM data chosen from a database of forensic cases (2001–2020) from the University of Milan, provided by the Laboratory of Forensic Anthropology and Odontology (LABANOF). The images were acquired as part of forensic judicial investigations and in accordance with the Italian Police Mortuary Regulation, approved to by the Milan prosecutor's office (Pubblico Ministero) and signed by the relatives and medical examiners. LABANOF received ethical approval from the Public Prosecutor (Milan), and the ethical committee of the University of Milan. Full ethical approval for image utilisation was gained from Liverpool John Moores University and the human participants (observers) provided informed consent (LJMU-UREC: 22/LSA/006). Only facial images were utilised in this research. To protect the identity of the subjects, the photographic material was not associated with any personal information, including name, date of birth, circumstances of death or associated pathologies.

### Main study

The identification study was carried out using a single-blind approach. The supervisory team (from LABANOF and Face Lab) selected subjects from a DVI database where the deceased had been identified previously. The supervisory team selected subjects from an archive of 105 unidentified Italian forensic cases and identified migrant cases, for which AM photographs of the face were available. The final 29 cases were over 18 years of age with at least one available AM and PM facial image. The supervisory team anonymised the data using alphanumeric code for each subject.

Since these images originated from real DVI casework involving migrants, they provide a realistic simulation, and the digital images associated with each subject reflected typical unstandardised AM images provided by relatives, social media or personal devices. The PM images reflected controlled images collected during PM examination or the recovery of the body.

The images were divided and organised into 2 folders:

1. AM folder—included subfolders corresponding to each of the 29 subjects containing between 1 and 10 facial images.
2. PM folder—included subfolders corresponding to each of the 29 subjects containing between 1 and 21 facial images.

The researcher followed the PM-to-AM facial image comparison protocol in an attempt to match AM targets (correct identities) to the PM subjects. 29 subjects represented a large number of facial image comparisons, as each PM subject was compared to every AM subject, making a total of 841 potential comparisons.

The PM-to-AM facial image comparison protocol and accompanying Excel spreadsheet were developed based on FISWG guidelines for facial comparison of living individuals [25, 29]. The FISWG protocol follows the Analysis, Comparison, Evaluation, and Verification (ACE-V) workflow employed for other forensic methods, such as fingerprint identification [28, 31]. The devised PM-to-AM protocol included the addition of a review phase to determine immediate exclusion of AM subjects before the comparison phase. In addition, the verification phase of ACE-V was not included in this experimental protocol, as peer review was considered unnecessary for an evaluation study. The protocol therefore included the following phases, which are described in the following sections:

- Phase 1.1—AM Image Analysis: subject factors, image factors and identifiable features recorded.
- Phase 1.2—PM Image Analysis: subject factors, image factors, identifiable facial features (either from the autopsy report or from visual analysis), decomposition stage and any other PM modifications recorded.
- Phase 2—Review: exclusion of non-matching AM subjects based on phase 1 records. Excluded AM subjects do not progress to phase 3.
- Phase 3—Comparison: PM-to-AM analysis to identify similarities and differences or to determine features that cannot be compared. Differences that may be due to PM changes or image factors recorded.
- Phase 4—Evaluation: an overall evaluation of the phase 3 records to establish the level of support for each potential candidate leading to match results.

Protocol guidance was provided in a pdf document containing classifications and reference images that relate to drop-down boxes in the Excel document. Each phase of the protocol has its own tab in the Excel chart. Example images and guidance can be found in the attached supplementary material.

Phase 1 included recording of subject and image factors and determination of identifying facial features for each PM and AM subject (see Tables 1, 2, 3 and 4).

**Table 1:** Example of Phase 1 recording of subject factors in ante-mortem (AM) or post-mortem (PM) analysis

CASE	GENDER (F/M/I)	SKIN (LIGHT/MEDIUM/DARK)	AGE (YOUNG/OLD)	NUMBER OF IMAGES	NOTES
AM001	M	LIGHT	YOUNG	3

**Table 2:** Example of Phase 1 recording of image factors in ante-mortem (AM) analysis

CASE	IMAGE	LIGHT EXPOSURE (1–3)	NOISE (1–3)	IMAGE VIEW	CAMERA DISTANCE	PHOTO DISTORTION	FACIAL MARKS
AM001	1	2	2	frontal	Close up	None	Mole on left cheek
AM001	2	2	2	Profile left	Extreme close up	None	As above
AM001	3	2	2	frontal	Full shot	None	As above
CASE	IMAGE	IMAGE ALTERATION	FACIAL EXPRESSION	IMAGE SOURCE	FACIAL ALTERATIONS	DISTRACTIONS	IMAGE QUALITY (1–6)
AM001	1	Colour correction	Smiling with teeth	Digital file	Piercing right ear	Hat	3
AM001	2	None	Neutral	Digital file	As above	None	4
AM001	3	None	Smiling—no teeth	Social Media	As above	Glasses	2

**Table 3:** Example of Phase 1 recording of image factors in post-mortem (PM) analysis

CASE	IMAGE	DENTAL (Y/N)	LOCATION	BLOATING (Y/N)	FACIAL MARKS
PM001	1	N	Mortuary/morgue	N	NONE
PM001	2	y	Mortuary/morgue	N	NONE
CASE	IMAGE	TRAUMA	OBSCURATION	FACIAL ALTERATIONS	DECOMPOSITION SCORE (A1-D4)
PM001	1	N	blood	Scar over left eye	A1
PM001	2	N	debris	Scar over left eye	A1

**Table 4:** Example of Phase 1 recording of individual features in ante-mortem (AM) or post-mortem (PM) analysis

CASE	FACIAL MARKS	DESCRIPTION	FACIAL MODIFICATIONS	DESCRIPTION	NOTES
AM001	Mole	Left cheek – beneath eye, level with nose	Pierced ears	Both ears – single hole	
AM001	Scar	Above right eyebrow – lateral end	Groomed eyebrows	Plucked	
PM001	Crease	Nasolabial creases—deep	Moustache	Handlebar	
PM001	Crease	Vertical glabella – deep, single	Pierced ear	Left – single hole	

Light Exposure was scored as 1 = well exposed, 2 = slightly under/over exposed, 3 = under/over exposed.

Noise was scored as 1 = no noise, 2 = moderate noise, 3 = high noise.

Image quality was scored as 1–6 and included:

1. Very good – excellent resolution, sharpness and illumination without obscuration or artifacts. Morphological analysis can be performed utilising small-scale features and detail.
2. Good—good resolution, sharpness and illumination with a maximum of 5% obscuration or artifact. Morphological analysis can be performed utilising the majority of small-scale features and detail.
3. Satisfactory – some deficit in resolution, sharpness or illumination with between 5–25% obscuration or artifact. Morphological analysis can be performed utilising some small-scale features and detail.
4. Sufficient – a number of deficits in resolution, sharpness or illumination with between 25–75% obscuration or artifact. Morphological analysis can be performed utilising only limited small-scale features.
5. Poor—significant deficits in detail, resolution, sharpness or illumination with between 75–80% obscuration or artifact. Morphological analysis can be performed utilising large-scale detail.
6. Insufficient – extremely poor detail, resolution, sharpness and illumination with more than 80% obscuration or artifact. Morphological analysis cannot be performed.

Decomposition was scored following published criteria [75] and included:

- A1 = Fresh
- B1 = Paler skin tone with skin slippage and some hair loss
- B2 = Gray to green discoloration with fresh flesh
- B3 = Brown discoloration at edges with drying of nose, ears and lips
- B4 = Purging of fluids from orifices with some bloating at neck and face
- B5 = Brown and black discoloration
- C1 = Collapse of flesh and tissues of eyes and throat
- C2 = Moist decomposition with bone exposure less than one half of the area
- C3 = Mummification with bone exposure less than one half of the area
- D1 = Bone exposure of more than half of the area with oils and decomposed tissue
- D2 = Bone exposure of more than half the area with desiccated or mummified tissue
- D3 = Bones largely dry but retaining some oils
- D4 = Dry bone

Phase 2 included a preliminary review of the phase 1 PM subject record with every phase 1 AM subject record to determine if any AM subjects can be excluded based on phase 1 subject factors (e.g., gender, age, skin tone) and/or identifying features (see Table 5). AM subjects not excluded progressed to phase 3 for each PM subject.

**Table 5:** Example of Phase 2 recording of post-mortem (PM) to ante-mortem (AM) review

COMPARISON	PM SUBJECT FACTORS	AM SUBJECT FACTORS	MATCH (Y/N)	ACTION (EXCLUDE/PROGRESS)	NOTES
PM001-AM001	M Light Young	F Light Old	N	Exclude	Gender, Age, Eyebrows

Phase 3 included PM-AM analysis following the FISWG feature list to identify similarities and differences and/or to determine features that could not be compared (e.g., due to image quality, obscuration and/or decomposition) between the PM subject and all its remaining AM subjects. Differences that may be due to PM changes or image factors were recorded (see Table 6).

**Table 6:** Example of Phase 3 recording of post-mortem (PM) to ante-mortem (AM) Comparison

PM CASE	AM CASE	FACIAL MARKS	FACIAL MODS	FACE SHAPE	HAIRLINE	EYES	CHEEKS	EYEBROWS
PM001	AM004	Similar mole on chin	Similar moustache and pierced left ear	Similar	Similar	Similar shape and tilt	Consistent	Similar shape and density
PM CASE	AM CASE	EARS	MOUTH	CHIN & JAWLINE	FACIAL HAIR	CREASES	CHALLENGES	NOTES
PM001	AM004	Similar	Similar lip shape	Similar	Similar	Nasolabial crease—similar pattern	PM changes	

Phase 4 included evaluation of phase 3 results to determine a level of support for each phase 3 AM subject being the target for the PM subject. The level of support refers to the degree of confidence that can be attributed to a match and measures the strength of the available evidence. The 5-point scale used for the level of support is adapted from Moreton [76] and includes:

1. *No support:* demonstrable differences that cannot be explained by PM changes, ageing or other facial alterations.
2. *Limited support:* broadly consistent facial features without identifying features or demonstrable differences. High levels of image deficiency, PM change or obscuration.
3. *Moderate support:* consistency across all features, but not all features can be compared due to image factors, PM changes or obscuration.
4. *Support:* consistency across all features, but difference/s that may be related to PM changes, ageing or other alterations or consistency across all features, but low or moderate quality image/s. Identifying features visible with low to moderate levels of detail.
5. *Strong support:* consistency across all features and identifying features, such as facial marks, facial modifications or scars.

Pairs scored as 1–4 (limited to strong) level of support for a match were recorded as a potential match. Pairs scored as 0 level (no) of support were recorded as non-matches (see Table 7).

**Table 7:** Example of Phase 4 recording of post-mortem (PM) to ante-mortem (AM) evaluation

PM CASE	AM CASE	DECOMP SCORE	OVERALL AM IMAGE QUALITY	OVERALL PM IMAGE QUALITY	IDENTIFYING FEATURES	LEVEL OF SUPPORT	NOTES
PM001	AM004	B3	4	3	Mole, moustache, pierced left ear, nasolabial crease pattern	Support (3)	Potential match
PM005	AM007	A1	2	2	Different nasal shape, dental pattern and hairline	No (0)	Non-match

The overall results were calculated as True Positives (number of target matches), True Negatives (number of non-target exclusions), False Positives (number of non-target matches) and False Negatives (number of target exclusions). The overall accuracy was calculated using the following formula [77]:

$$\text{Accuracy rate}= \frac{\text{True Positives}+\text{True Negatives}}{\text{True Positives}+\text{True Negatives}+\text{False Positives}+\text{False Negatives}}$$

### Inter-observer study

To assess the reliability of the facial image comparison protocol, a selection of 15 PM-AM pairs from the main study was analysed by 3 different practitioners with varying levels of experience: one newly trained, one with more than 2 years’ experience and one with 30 years’ experience. All practitioners were blind to the target identities. The practitioners were provided with the necessary protocol tables and guidance documents and were instructed to perform facial image comparison (excluding phase 2) for each of the PM-AM pairs following the same protocol as for the main study.

The results of the three practitioners were analysed using descriptive statistics, including calculation of accuracy rate and false and true positive and negatives [77]. The inter-observer reliability was calculated using the Interclass Correlation Coefficient (ICC) for the absolute agreement while Kendall’s Coefficient (W) was used to determine the degree of agreement among observers [78] (Tables 8, 9 and 10).

**Table 8:** The results of phase 4 evaluation of post-mortem to ante-mortem (PM-AM) facial image comparisons between each PM subject and the AM subjects progressed from phase 3

PM	AM selected after Facial Review Phase	Level of Support	AM target	PM	AM selected after Facial Review Phase	Level of Support	AM target
A	8	No	14	N	9	No	5
	14	Moderate			24	No	
	4	No			5	Strong	
AA	12	No	27	O*	14	Limited	20
	27	Strong			20	Support	
	28	No		P*	24	Support	24
AB*	3	Limited	29		9	Limited	
	29	Limited		QM	10	No	4
	23	Moderate			1	No	
AZ	28	Strong	28		2	No	
BS*	4	No	10		4	Support	
	18	No		R	24	No	16
	10	Moderate			9	No	
	1	Limited			16	Moderate	
	4	No		S	10	No	2
C	21	Limited	21		2	Support	
DS	17	Support	17		4	No	
E	29	No	3		6	No	
	3	Support		T*	14	Limited	18
F	3	No	15		18	Strong	
	7	No		U*	10	No	6
	15	Moderate			1	Moderate	
G	12	Support	12		2	No	
	27	No			6	Strong	
	28	No		V	13	Strong	13
H	19	Limited	19		2	No	
I	11	Strong	11	W#	18	No	1
JO	22	Support	22		10	Limited	
K#	15	No	23	X	14	No	8
	7	No			1	No	
	19	Limited			8	Strong	
	23	No		YT	5	No	9
L	25	Strong	25		20	No	
M	19	No	7		24	No	
	7	Strong			9	Moderate	
				Z	26	Strong	26

^#^ = PM cases incorrectly identified. * = PM cases matched to more than one AM subject

**Table 9:** Post-mortem to ante-mortem (PM-AM) facial image comparison exclusions and potential matches for non-targets and targets

	AM Non-targets	AM targets	Total
Exclusion	34 (97.1%)	2 (7%)	29
Potential Match	9 (25%)	27 (93.1%)	35
	43	29	72

**Table 10:** Level of support recorded by each observer for each post-mortem to ante-mortem (PM-AM) comparison case

PM	AM	OBSERVER 1	OBSERVER 2	OBSERVER 3	AM TARGET
A	4	No support	Limited support	No support	14
C	21	Support	Moderate support	Limited support	21
DS	17	Support	Support	Support	17
F	7	No support	No support	No support	15
I	11	Strong support	Support	Strong support	11
M	19	No support	Limited support	Limited support	7
N	24	Moderate support	Moderate support	No support	5
O	20	Limited support	Limited support	Limited support	20
QM	1	No support	Limited support	No support	4
T	18	Strong support	Support	Strong support	18
T	14	No support	Limited support	Limited support	18
W	18	No support	No support	No support	1
YT	9	Limited support	Moderate support	Moderate support	9
YT	5	No support	No support	No support	9
Z	26	Strong support	Moderate support	Strong support	26

True positives (correct identifications – rated limited to strong support) and true negatives (correct exclusion – rated no support) are shaded grey

## Results

### Main study

In the AM folder, there were 12 female-presenting subjects (41%), and 17 male-presenting subjects (59%). 15 subjects were categorised as having light skin (52%) and 14 subjects as having dark skin (48%). The majority of the subjects (52%) presented one or two images for comparison. There were 98 AM images in total, with the majority (51%) of moderate quality (4–5), 34% of high quality (1–3) and 15% insufficient quality (6) for comparison. Significantly, given the importance of social media for image collection and the widespread use of camera and smartphone filters, 26% of AM images presented image alterations, with photo-retouching (15%) and filters (8%) recorded most frequently.

In the PM folder, there were 11 female-presenting (38%) and 17 male-presenting (59%) subjects, with one subject in the PM sample classified as indeterminate gender (3%). 15 subjects were categorised as having light skin (52%) and 14 subjects as having dark skin (48%). 76% of the PM subjects were assigned an A1 decomposition score, indicating that most of the PM subjects showed a high degree of facial preservation. No subject scored lower than a B5 decomposition score.

In total 71 full PM-AM facial image comparisons were carried out, with the majority (82–96%) of AM subjects excluded at the review phase for each PM subject. One AM target was excluded at the review phase. The average number of potential matches to an individual PM subject was 2.5, with eight PM subjects recording three potential matches and seven PM subjects recording two potential matches at the review phase. The maximum number of potential matches to an individual PM subject was five and the minimum number was one.

The true positive rate was 93.1% (27 of the 29 AM targets were matched to the correct PM subject) and the true negative rate (specificity) was 79.1% (34 of the 43 non-targets were excluded). The false positive rate was 21% (9 of the 43 non-targets were matched to PM subjects) and the false negative rate was 7% (2 of the 29 AM targets were excluded). Therefore, the overall accuracy rate was 84.7%.

After phase 4, the PM subjects were recorded with between 1–3 potential matches, with 79.3% (*n* = 23) recording only one potential match and only one PM subject (3.4%) recording three potential matches. If an identification decision had been made based on the AM subject that received the highest level of support for a potential match at each PM subject, three of the PM subjects would have been incorrectly identified (false positives). This would produce a true positive rate of 89.7%, a true negative rate of 93%, a false positive rate of 6.9%, a false negative rate of 10.3% and an accuracy rate of 91.7%. For the PM subject who received three potential matches after phase 4 (the target and two non-targets), the target received a lower level of support (limited) than one of the non-targets (moderate). However, in a real DVI situation, the target would not be excluded, and confirmation of identity would be attempted using primary identifiers where possible for all potential matches.

After phase 4, 72.4% of the PM subjects (*n* = 21) were only matched to the targets (no non-targets as potential matches). In total, 71.4% of the true positives were recorded with high levels of support (support or strong support), whilst 29.6% were recorded with low levels of support (limited or moderate). The false positives were all recorded with low levels of support (limited or moderate). In the two cases where false negatives (targets excluded) were recorded, the non-target matches were scored at the lowest level of support (limited). One target was excluded in the review phase due to facial mark inconsistency (tattoo and mole present at PM and not visible AM). The other target was excluded at the evaluation phase and this subject presented only one AM image and did not have any visible identifying marks. Both excluded targets recorded low numbers of AM images (1–2) with low image quality and one image presented alterations.

Subjects with a low number of AM images available were more likely to receive an inconclusive level of support. This contributed to both false negatives and false positives. The highest level of support was recorded more frequently where the AM subject had at least one high-quality image.

During the comparison phase, it was noted that inherent PM changes made certain features less useful for analysis than in living facial image comparison. For example, in the majority of comparisons, assessment of the eyes or skin colour was not possible, and photo-retouching negatively affected the assessment of skin colour. In addition, nasal shape appeared different between AM and PM images for a number of subjects, even when other identifying features suggested high levels of support for a match; the PM nasal tip shape appeared more downturned than in the AM images.

### Inter-observer study

In the large majority of cases (80%), all three practitioners recorded similar levels of support with only one level distinction between practitioners.

The inter-observer reliability was calculated using the Intraclass Correlation Coefficient (ICC) and Kendall's Coefficient of Concordance. The ICC two-way mixed model on absolute agreement was used to analyse inter-observer reliability for the level of support expressed by all 3 observers [79]. The values of the ICC range from 0 to 1, with a higher value signifying better reliability. ICC less than 0.40 was considered poor, between 0.40–0.59 as fair, 0.60–0.74 as good, and greater than 0.75 as very good [79]. The ICC was 0.813 with a 95% confidence interval between 0.620–0.923, which is considered a good inter-observer reliability score. In addition to the absolute agreement, Kendall’s Coefficient of Concordance (W) was calculated to take into account the degree of agreement among the three observers [80]. In other words, the test helps to differentiate between levels of supports with only one point of difference versus not close at all. Kendall’s coefficient ranks from 0 to 1 with higher values close to 1 highlighting a strong inter-observer agreement. Kendall’s coefficient was found to be 0.885, which suggests good inter-observer agreement.

Practitioner/observer 1 recorded 100% true positives, 87.5% true negatives, 6.7% false positives and 0% false negatives. The overall accuracy rate was 93.3%. Practitioner 1 had the most experience (30 years) in facial image comparison.

Practitioner/observer 2 recorded 100% true positives, 37.5% true negatives, 33.3% false positives and 0% false negatives. The overall accuracy rate was 66.7%. Practitioner 2 had moderate (two years) experience in facial image comparison and was the most cautious in the choice of level of support, never recording the highest level of support (4) and less likely to record no support (0).

Practitioner/observer 3 recorded 100% true positives, 75% true negatives, 13.3% false positives and 0% false negatives. The overall accuracy rate was 86.7%. Practitioner 3 was newly trained in facial image comparison.

Overall, the practitioners/observers recorded 100% true positives, 67% true negatives, 17.8% false positives and 0% false negatives, and the overall accuracy rate was 82.2%.

## Discussion

These preliminary results suggest that, following the developed protocol, PM-to-AM facial image comparison can be reproducible and reliable across practitioners with varying degrees of previous experience. The combined practitioner identification rates on the inter-observer test sample were similar to the identification rates produced by a single researcher in the main study, with slightly higher true positives (100% > 93.1%) and slightly lower true and false negatives (67% < 79.1% and 0% < 7% respectively) and false positives (17.8% < 21%). The overall accuracy rates were similar for combined practitioners (82.2%) and the single researcher (84.7%). The researcher had never performed facial image comparison prior to carrying out this research. Where practitioners disagreed on cases, it was usually due to cautious evaluation at phase 4 by one practitioner. All practitioners independently agreed on the final outcomes and reached ceiling levels for true positives and false negatives. Implementing a simplified 4-point scale of support (No; Inconclusive; Moderate; Strong) could prove beneficial for inexperienced practitioners where there are multiple PM-AM comparisons.

The majority of AM images were recorded as moderate quality (4–5) and correct matchings were possible with an accuracy rate of 84.7%, a specificity rate of 79.1% (true negatives) and a sensitivity rate (true positives) of 97%, implying that the method correctly identifies matches most of the time but finds exclusion more difficult to achieve. The false positive rate was 20.9% and the false negative rate was 7%. In most forensic identifications, it is crucial to maintain low false positive rates as misidentification can lead to severe legal consequences [81]. However, in DVI scenarios it is more important to maintain a low false negative rate than a low false positive rate, as keeping non-targets in the pool of potential matches will lead to fewer misidentifications than excluding the target. Therefore, our low false negative rate is encouraging for the use of the protocol in PM identification. However, any target exclusions will lead to, at best, delays to the identification process and, at worst, misidentification. The key factors affecting false positive/negative rates appear to be poor AM image quality and low numbers of AM images. When AM image quality is low and less facial feature detail is visible, more matches will be possible, increasing the false positive rate, but some distinguishing features noted in PM images may not be visible leading to an additional increase in false negatives. This is consistent with facial image comparison evaluation studies for living individuals [26]. Interestingly, for early-stage (A1-B5) decomposition, the degree of decomposition does not appear to affect the accuracy of the facial image comparison.

The inclusion of a review phase (phase 2) to the protocol enabled rapid exclusion of non-targets, but also led to a target exclusion. The researcher utilised recorded subject factors and identifying features in this phase, and inconsistent features (tattoo and mole) between AM and PM images were recorded as justification for the exclusion. This result suggests that a review phase should only consider subject factors (such as gender presentation, broad skin tone and age), so that a more detailed analysis of identifying features is possible in phase 3.

These results suggest that some facial features should not be considered for PM-AM comparisons due to immediate PM changes even with fresh bodies including the eyes and skin colour. A similar observation was documented in a previous study [26] of living subjects, which mentioned unreliable skin colour and luminance in unstandardised facial images. Similarly, researchers [44] have highlighted unreliable skin colour due to post-production filters that can lighten and smooth the skin appearance in unstandarised AM images. The challenges around PM eye assessment have also been noted previously [52] where both shrinkage and bloating at the orbits has been recorded in deceased individuals. Additionally, in a further PM study [17] where groups of students and professionals were asked to identify the most useful features after completing a PM recognition task, the eyes were never selected as a useful feature. In this previous PM study, the nose was selected as one of the most useful features, but our study suggests that there may be significant changes to nasal tip shape due to pressure caused by a body bag.

True positives with strong support were associated with the presence of one or multiple identifying features, in particular facial marks. At the same time, lower support levels were generally associated with a very limited to no identifying features. This is in line with previous research highlighting the importance of identifying features for PM-AM image comparison, even with unstandardised images [82] or images with a significant age difference.

The most commonly recorded identifiers were facial marks, followed by ears, scars, teeth and piercings. Facial marks have been established as important for identification in relation to living individuals [55, 56], in facial recognition systems [83, 84] and as secondary identifiers for DVI procedures [57, 85]. This finding also aligns with existing literature that highlights the uniqueness of the external ear morphology and its use as a biometric for living [34, 68] and deceased individuals [73]. Dental pattern in smiling face images has previously been shown to be a vital feature for the identification of the deceased [86–89]. However, there was limited availability of AM and PM dental images in the sample used for our study.

This pilot study evaluates a relatively small cohort of post-mortem faces, but the size of the evaluation was significant (a total of 841 potential comparisons). In this way, the study represents a real DVI scenario with a missing persons list and multiple casualties.

## Conclusions

PM-AM facial image comparison following a defined protocol can be used to identify potential matches, even when the PM subjects show early post-mortem changes. This could be particularly useful in large-scale disasters and deceased migrant identification, where primary identifiers cannot be utilised, and secondary identifiers can provide enough evidence for identification (as approved by the relevant legal authority). Facial features such as moles, scars, ears, teeth and deliberate modifications have been shown to be particularly relevant when conducting a facial image comparison and their presence can lend strong support to both exclusions and potential matches. The protocol was found to be reliable with good agreement between practitioners.

Recommendations for PM-AM facial image comparison include:

- Utilising only recorded subject factors for exclusion at the review phase (phase 2).
- Introducing a 4-point scale of support for a potential match, including no support (0), inconclusive (1), moderate support (2) and strong support (3).
- Excluding analysis of the eyes, nasal tip shape and skin colour from phase 3.
- Focusing on highly distinctive features such as marks, ears, teeth and modifications.

The team further recommend the use of three-dimensional (3D) scan technology (Computed Tomography or surface scanners) at the post-mortem examination stage to collect data of unknown bodies to facilitate any facial morphology assessments. Further research is necessary when the subject shows more advanced stages of decomposition, involving a larger sample size and the use of 3D imagery to record the PM faces.

The protocol guidance and practical recording chart are provided for practitioner use in attached supplementary materials A and B.

## Supplementary Information

Below is the link to the electronic supplementary material.

Supplementary file1 (DOCX 3838 KB)

Supplementary file2 (XLSX 118 KB)

## Data Availability

The data that support the findings of this study are not publicly available due to the sensitive nature of the data. Access from the Laboratory of Forensic Anthropology and Odontology (LABANOF) at the University of Milan includes restrictions in accordance with the Italian Police Mortuary Regulation and requires approval by the Milan prosecutor's office (Pubblico Ministero), the medical examiners and the related relatives.

## Footnotes

1 https://www.interpol.int/en/How-we-work/Forensics/Disaster-Victim-Identification-DVI
