# Correction to: Post-mortem to ante-mortem facial image comparison for deceased migrant identification

**DOI:** 10.1007/s00414-024-03325-w

**Published:** 2024-09-07

**Authors:** Caroline Wilkinson, Martina Pizzolato, Danilo De Angelis, Debora Mazzarelli, Annalisa D’Apuzzo, Jessica Ching Liu, Pasquale Poppa, Cristina Cattaneo

**Affiliations:** 1 Face Lab, Liverpool John Moores University, Liverpool, UK; 2 LABANOF, University of Milan, Milan, Italy

**Correction to: International Journal of Legal Medicine (2024)**

10.1007/s00414-024-03286-0

Originally, the paper was published with error. There is a typo on page 10 in the Results section, the Main study. The second-to-last sentence of the first paragraph should be revised to read:

“This would produce a true positive rate of 89.7%, a true negative rate of 93%, a false positive rate of 6.9%, a false negative rate of 10.3% and an accuracy rate of 91.7%”.

The original article has been corrected.

