# Supplementary material for: Post-mortem to ante-mortem facial image comparison for deceased migrant identification
Source: Int J Legal Med. 2024 Aug 16;138(6):2691–706. doi: 10.1007/s00414-024-03286-0 (PMC11490436; doi:10.1007/s00414-024-03286-0)
Supplement: Supplementary file 1 — Supplementary file1 (DOCX 3838 KB) [file 414_2024_3286_MOESM1_ESM.docx]

**Post-mortem to ante-mortem facial image comparison for deceased migrant identification**

Caroline Wilkinson^1^, Martina Pizzolato^1^, Danilo De Angelis^2^, Debora Mazzarelli^2^, Annalisa D’Apuzzo^2^, Jessica Ching Liu^1^, Pasquale Poppa^2^ and Cristina Cattaneo^2^

^1^ Face Lab, Liverpool John Moores University

^2^ LABANOF, University of Milan

Corresponding author: [c.m.wilkinson@ljmu.ac.uk](mailto:c.m.wilkinson@ljmu.ac.uk) ORCID: <https://orcid.org/0000-0002-4603-5554>

# Scope

This protocol provides a detailed, step-by-step guide for using the PM-to-AM Facial Image Comparison Recording chart, created to aid decision-making during post-mortem to ante-mortem (PM-to-AM) morphological facial comparison.

It is important to note that PM-to-AM Facial Image Comparison is primarily used for identity exclusion and should not be relied upon as the sole means of identification.

To utilise this protocol, practitioners should:

- have access to images of unidentified deceased individuals (PM case) and missing persons (AM case).
- have access to the " PM-to-AM Facial Image Comparison" Excel spreadsheet.
- possess knowledge of facial anatomy, PM changes, environmental factors, and the impact of decomposition on facial appearance.
- be familiar with the resources and guidelines outlined in Table 1 and Table 2.

# Structure

The protocol and accompanying spreadsheet are structured following FISWG guidelines, which were initially developed for facial comparison of living individuals. The protocol employs the ACE-V (Analysis, Comparison, Evaluation, and Verification) workflow, which is preceded by a holistic review of the face.

The various sections of the protocol correspond to the different sections of the PM-to-AM Facial Image Comparison spreadsheet, organised as follows:

- **AM List**
- **PM List**
- **Phase 1 - AM Analysis** (equivalent to Analysis in the ACE-V): In this phase, AM images are analysed against specific image factors.
- **Phase 1 - PM Analysis** (equivalent to Analysis in the ACE-V): In this phase, the decomposition stage and other PM modifications are recorded, and any obstruction or trauma is noted.
- **Phase 2 - Facial Review**: In this phase, individuals who are clearly not a match based on broad biological profile (age, gender, skin tone) will be eliminated, and those who are a possible match will progress onto the phase 3. Where these factors are uncertain the individual will not be eliminated.
- **Phase 3 - Comparison** (equivalent to Comparison in the ACE-V): In this phase, images of potential matches are analysed, following FISWG recommendations. Each feature is analysed to identify similarities and differences, and any features that cannot be compared (due to image limitations) are noted. Differences that may be due to PM changes, image factors or age differences are noted here.
- **Phase 4 - Evaluation** (equivalent to Evaluation in the ACE-V): This phase involves an overall evaluation of the results obtained during phase 3 to determine the level of support for a match.
- **Phase 5 - Verification^a^** (equivalent to Validation in the ACE-V): This phase involves a review of the entire process and results by an independent peer reviewer.

^a^ There is not a specific dedicated Excel section for this step.

# Recommendations:

- If dentition is visible in both AM and PM images, an odontologist should be contacted for identification.
- Post-mortem facial comparison can offer significant advantages in several situations, for example:
  - When there is a substantial number of deceased individuals and missing persons with facial images available.
  - When resources for conducting extensive DNA testing on victims and their relatives are limited or where DNA collection is not possible from AM or PM sources.
  - When access to primary identification data such as DNA, fingerprints, and radiographs is restricted.
  - When the genetic makeup of the deceased is of limited use due to kinship between victims.

**Table 1**. References documents.

| Title of the resource | Content | Author | Available at |
| --- | --- | --- | --- |
| Facial comparison overview and methodology guidelines | Standard guide illustrating the required steps of comparison - Analysis, Comparison, Evaluation and Verification (ACE-V). | FISWG | <https://fiswg.org/fiswg_facial_comparison_overview_and_methodology_guidelines_V1.0_20191025.pdf> |
| Facial Image Comparison Feature List for Morphological Analysis | This document provides a list of 19 facial components to be taken into consideration during a facial comparison. It is not intended as a classification or atlas, but rather as a comprehensive collection of features that should be examined. | FISWG | <https://fiswg.org/FISWG_Morph_Analysis_Feature_List_v2.0_20180911.pdf> |
| Image factor to consider in facial image comparison: | This document explains the primary factors that can affect the quality of an image and the visibility of facial features. | FISWG | <https://fiswg.org/fiswg_image_factors_to_consider_in_facial_img_comparison_v1.0_2021.05.28.pdf> |
| Physical stability of facial features of adults | This guide aims to assist in the interpretation of anticipated and unexpected changes in visible facial features. | FISWG | <https://fiswg.org/fiswg_physical_stability_of_facial_features_of_adults_v2.0_2021.05.28.pdf> |
| Best Practice Manual For Facial Image comparison | This document outlines the procedures and protocols for conducting photographic facial comparison, including a comprehensive overview of the ACE-V workflow. Appendix B provides detailed descriptions and examples of factors that can influence facial appearance. | ENFSI | <https://enfsi.eu/wp-content/uploads/2017/06/ENFSI-BPM-DI-01.pdf> |
| Facial Image Comparison Best Practices for Markups and Annotations | This document aims to offer guidance and best practices for the use of markups and annotations in the process of facial image comparison. | FISWG | <https://fiswg.org/fiswg_facial_image_comp_best_practices_markups&annotations_v1.0_2021.05.28.pdf> |

**Table 2.** Other reference documents

- Bacci, N., Houlton, T.M.R., Briers, N. and Steyn, M. (2021b) Validation of forensic facial comparison by morphological analysis in photographic and CCTV samples. International Journal of Legal Medicine, 135 (5), 1965-1981
- Caplova Z., Obertova Z., Gibelli D.M., Mazzarelli D., Fracasso T., Vanezis P., Sforza C. and Cattaneo C. (2017) The Reliability of Facial Recognition of Deceased Persons on Photographs. J Forensic Sci. 62(5):1286-1291. doi: 10.1111/1556-4029.13396. Epub 2017 Feb 15. PMID: 28205214.
- Caplova, Z., Gibelli, D.M., Poppa, P., Cummaudo, M., Obertova, Z., Sforza, C. and Cattaneo, C. (2018a) 3D quantitative analysis of early decomposition changes of the human face. International Journal of Legal Medicine, 132 (2), 649-653
- European Network of Forensic Science Institutes (ENFSI) (2018) Best Practice Manual for Facial Image Comparison. Version 01. [online] Available at: http://enfsi.eu/wp-content/uploads/2017/06/ENFSI-BPM-DI-01.pdf
- Megyesi, M.S., Nawrocki, S.P. and Haskell, N.H. (2005) Using accumulated degree-days to estimate the postmortem interval from decomposed human remains. Journal of Forensic Sciences, 50 (3), 618-626.
- Schüler, G. and Obertová, Z. (2020) Visual identification of persons: Facial image comparison and morphological comparative analysis. In: (ed.) Statistics and Probability in Forensic Anthropology. pp. 313-330
- Wilkinson, C. and Tillotson, A. (2012) Post-mortem prediction of facial appearance. In: Wilkinson, C. and Rynn, C (eds) Craniofacial Identification. Cambridge: Cambridge University Press. pp. 166-183

# Ante-mortem (AM) List

Access the PM-to-AM Facial Image Comparison Excel spreadsheet and navigate to the **AM List** tab**.** Once there, follow the below instructions to complete the columns.

| a.1 AM code |
| --- |
| Insert the ante-mortem case identifier code |

| a.2 Presenting gender | |
| --- | --- |
| Select the presenting gender that best describes the individual using the list below. | |
| F | Female: presents physical gender expression that are generally typical of female individuals. This might include facial features, hairstyles, makeup, clothing. |
| M | Male: presents physical gender expression that are generally typical of male individuals. This might include facial features, hairstyles, facial hair, clothing. |
| I | Indeterminate: presents physical traits that dot not fit into the traditional binary categories of female and male. |

| a.3 Skin tone | |
| --- | --- |
| Select the skin tone that best describes the individual in the pictures using the list below. If the skin tone cannot be ascertained with any degree of certainty, please leave this column empty. | |
| Light | Pale or fair complexion |
| Medium | Warm brown complexion |
| Dark | Deep brown to black complexion |

| a.4 Estimated age | |
| --- | --- |
| This description should be used if the age of the missing individual is unknown. If neither category applies, please leave this column empty.  If the age is known, please make a note in the a.6 Notes section. | |
| Young adult | The individual appears to be late teenage to young adulthood. |
| Old adult | Individual that physically appear to be above 70 years of age. The individual might present gray hair or balding, skin texture changes (wrinkles, age spots, leathery skin etc..) and low levels of facial fat. |

| a.5 Images available |
| --- |
| Insert the total number of images available. |

# Post-mortem (PM) List

Access the "PM-to-AM Facial Image Comparison " Excel spreadsheet and navigate to the **PM List** tab**.** Once there, follow the below instructions to complete the columns.

| b.1 PM code |
| --- |
| Insert the post-mortem case identifier code |

| b.2 Presenting gender/ sex | |
| --- | --- |
| Select the presenting gender that best describes the individual or biological sex (if known from the autopsy report) using the list below.  ⚠*The biological sex reported in the autopsy report might not be representative of the living presented gender. Use the b.6 Notes to add any relevant information.* | |
| F | Female: presents physical gender expression that are generally typical of female individuals. This might include facial features, hairstyles, makeup, clothing. |
| M | Male: presents physical gender expression that are generally typical of male individuals. This might include facial features, hairstyles, facial hair, clothing. |
| I | Indeterminate: presents physical traits that dot not fit into the traditional binary categories of female and male. |

| b.3 Skin tone | |
| --- | --- |
| Select the skin tone that best describes the individual using the list below. If the skin tone cannot be ascertained with any degree of certainty, please leave this column empty. | |
| Light | Pale or fair complexion |
| Medium | Warm brown complexion |
| Dark | Deep brown to black complexion |

| b.4 Estimated age | |
| --- | --- |
| This description should be used if the age of the deceased individual is unknown. If neither category applies, please leave this column empty.  If the age is known, please make a note in the a.6 Notes section. | |
| Young adult | The individual appears to be late teenage to young adulthood. |
| Old adult | Individual that physically appear to be above 70 years of age. The individual might present gray hair or balding, skin texture changes (wrinkles, age spots, leathery skin etc..) and low levels of facial fat. |

| b.5 Images available | |
| --- | --- |
| Indicate the number of available images selecting the appropriate categories.  ⚠ *If image quality is low or images are blurred, document it in the b.6 Notes* | |
| <10 or 1to3 | Less than 10 PM images |
| 4 to 9 | Between 4 and 9 PM images |
| 10+ | More than 10 PM images |

| a.6 Notes |
| --- |
| Insert any additional information relevant to the PM case |

# Phase 1 – AM Analysis

Access the PM-to-AM Facial Image Comparison Excel spreadsheet and navigate to the Phase 1-AM **Analysis** tab**.** Once there, follow the below instructions to complete the columns.

In this stage, you are required to analyse the quality and suitability of the AM images for facial comparison against guided image factors. You are not required to assess the facial morphology.

The factors you will consider include lighting, obstructions and quality of the images. The latter in particular plays a crucial role in facial comparison as it influences the strength and level of confidence of the final result.

To minimise the confirmation bias, the practitioner **should** analyse AM and PM images separately.

| 💡︎ Before starting, familiarise with the image factor concepts explained in “Image factor to consider in facial image comparison” and “Best Practice Manual for Facial Image Comparison” - see Table 1 on how to access the material. |
| --- |

| 1.1 AM case |
| --- |
| Insert the ante-mortem case identifier code |

| 1.2 IMG file |
| --- |
| Insert the ante-mortem image file name |

| 1.3 Image view | |
| --- | --- |
| Frontal (F) | Right and left ears are visible (or the area where the ear would be, if covered by hair). The chin and forehead are more or less visible. |
| Oblique Right (OR)  Oblique Left (OL) | One ear (or the area where the ear would be if covered by hair) and one side of the jaw are apparent, while a portion of the other side of the face is partially obscured. Commonly known as a three-quarter view. |
| Profile Right (PR)  Profile Left (PL) | One ear (or the area where the ear would be if covered by hair) one side of the jaw and facial profile are visible. The opposite side is completely obscured. Commonly known as a profile view. |

| 1.4 **Camera distance** |
| --- |
| Where the camera is positioned in relation to the subject.  Select from the relevant drop-down list. |
| 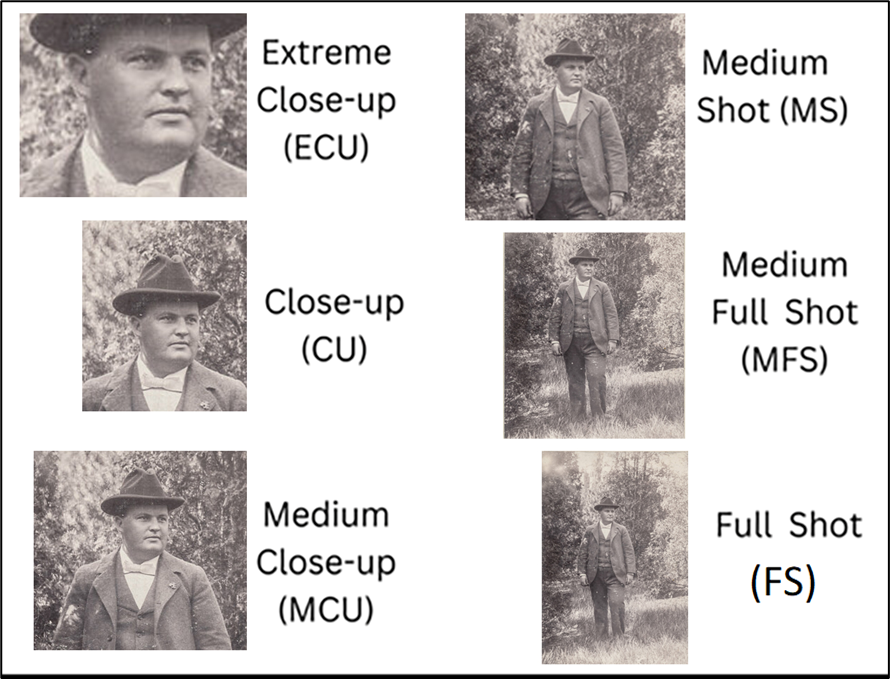  Figure 2. Distance camera-subject with examples ["Man standing in a field" by simpleinsomnia is licensed under CC BY 2.0. To view a copy of this license, visit <https://creativecommons.org/licenses/by/2.0/?ref=openverse>] |

| 1.5 **Image source** | |
| --- | --- |
| Original printed photo (OP) | Original printed photograph |
| Scanned print (SP) | Printed scan of original photograph |
| Scanned copy (SC) | Scan of a non-original paper photograph |
| Original File (OF) | Original digital file (usually .jpg, .png or .tiff) |
| Digital File (DF) | Digital file, but unsure if it is the original |
| Social media image (SM) | Image downloaded from social media |
| Picture of picture (PP) | Image copy of a digital or paper photograph |
| Screenshot (SS) | Screenshot or video still |

| 1.6 Facial Marks |
| --- |
| Any type of permanent or transient skin mark, including spots, moles, birthmarks, blemishes and freckles. Insert a description of any observed facial marks.  ⚠ *In some cases image artifacts (due to lighting, quality etc…) might be mistaken for facial marks. State it in the description when you unsure about the nature of the facial mark observed.* |

| 1.7 Facial Alterations |
| --- |
| Any type of permanent skin modification, including scars, tattoos (tattooed eyebrows and lips included), implants and piercings. Insert a description of any observed alteration.  ⚠ *In some cases image artifacts (due to lighting, quality etc…) might be mistaken for scars. State it in the description when you unsure about the nature of the mark observed.*  ⚠ *In some cases individuals might be wearing fake piercings in a way that mimic the appearance of real piercings.* |

| 1.8 Photographic Distortion | | |
| --- | --- | --- |
| Photographic distortion refers to the way an object can appear warped due to perspective, camera lens type or post production manipulation (see figure 3). | | |
| Type | Description | Score |
| Normal face | No visible facial distortion (see figure 3a) | No |
| Barrel | Horizontal lines appear curved. The central features of the face appear expanded (see figure 3d) | Yes |
| Pin-cushioning | Horizontal lines appear curved. The central features of the face appear squeezed (see figure 3e) |  |
| Thinner | Aspect-ratio changes producing horizontal squeeze (see figure 3b) |  |
| Wider | Aspect-ratio changes producing vertical squeeze (see figure 3c) |  |


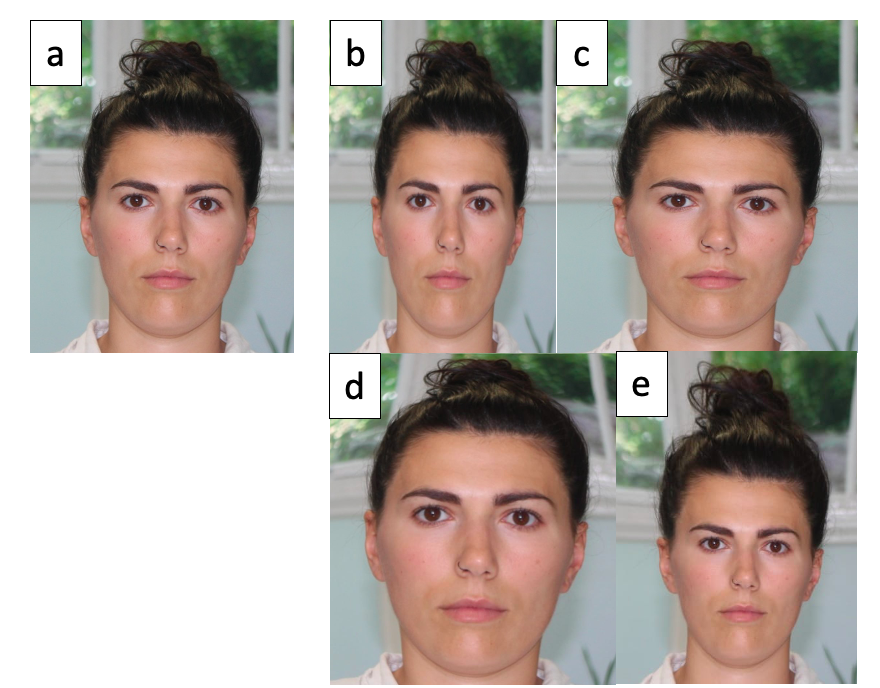


**Figure 3.** Image with no distortion (a) and some common photographic distortions (b-e)

| 1.9 Light Exposure | | |
| --- | --- | --- |
| Light exposure may influence the visibility of some facial features (see figure 4). | | |
| Exposure intensity | Description | Score (Figure 4) |
| Well exposed | Balanced exposure with no excessively dark or bright areas. All features should be visible (see figure 4.1). | 1 |
| Slightly overexposed  Slightly underexposed | Some exposure imbalances with areas that are more difficult to distinguish (see figures 4.2a and 4.2b) due to brightness or shadow. | 2 |
| Overexposed  Underexposed | Exposure imbalance with very bright or very dark areas and difficult or indistinguishable details (see figures 4.3a and 4.3b). | 3 |

**Figure 4.** Different levels of photographic exposure.

1 = balanced; 2 = slight imbalance; 3 = greater imbalance; A = overexposed; B = underexposed

| 1.10 Noise | | |
| --- | --- | --- |
| Noise refers to the spots or grain in the image due to low light, camera settings or, in some cases, deliberate post-production filters (see figure 5).  ⚠*Noise is different from pixel. Noise appears as random spectacles, while pixels are the unit of an image visible as small-scale squares.*  *⚠In some cases, high noise levels can make identification of facial marks difficult. Note any relevant observation in the 1.15 Note or in the 1.6 Facial marks boxes.* | | |
| Level of noise | Description | Score (Figure 5) |
| Low | No visible grain. Pixelation may be visible with low quality images. | 1 |
| Medium | Some fine grain, but facial details and marks on the skin are visible. | 2 |
| **High** | Numerous grains covering the whole image, with a lack of fine detail. Facial marks may be difficult to observe. | 3 |

*
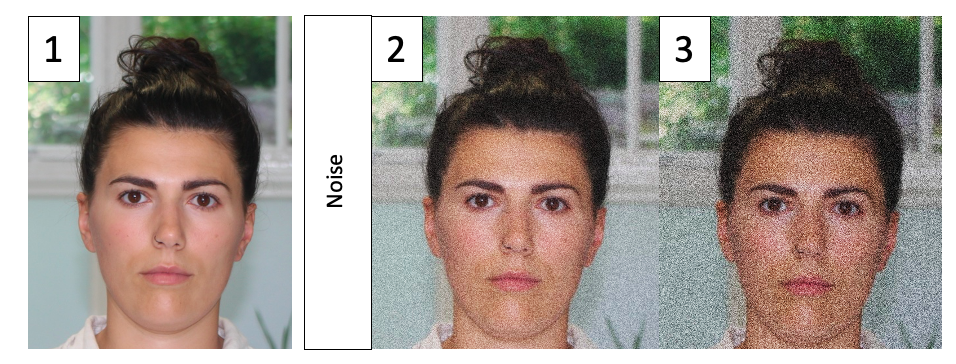
*

**Figure 5.** Examples of different type of noise that can affect an image.

| 1.11 Image alteration | |
| --- | --- |
| Image alteration refers to photo-editing processes to change appearance.  Select the appropriate choice using the drop-down list. | |
| Alteration | Description |
| None | No obvious manipulation (see figure 6a) |
| Colour correction | Changes to saturation, intensity and other levels (see figure 6f) |
| Photo-retouching | Skin retouching or use of social media filters; teeth whitening; background removal (see figure 6g) |
| Other filters | Any other social media or app filters (see figure 6h) |
| Multiple | Presenting multiple alterations |

**
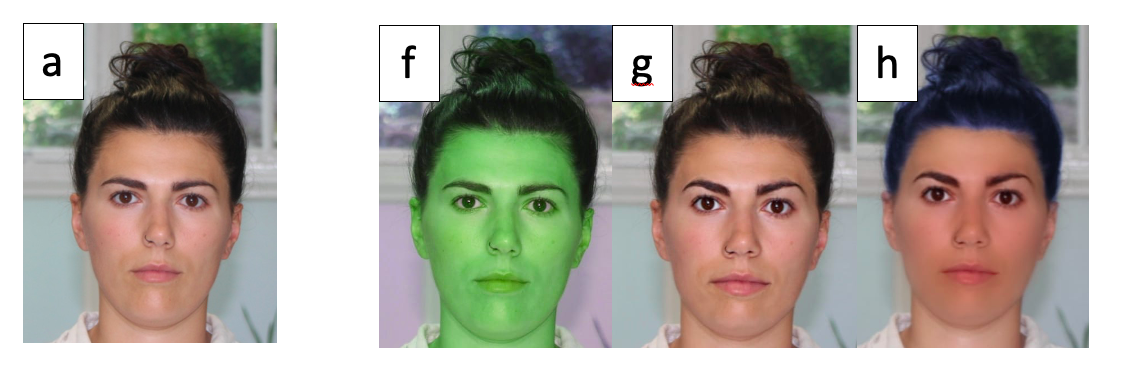
**

**Figure 6.** Examples of photo-editing: colour misrepresentation [f]; photo-retouching [g]; multiple alterations [h].

[a] original photograph with no alteration

| 1.12 Distractions | |
| --- | --- |
| Some facial features may be obscured.  Select using the drop-down list.  ⚠*If other elements such as lighting, head pose etc… are obscuring some features this should be noted in the relevant section and a detailed descriptions of what features are obscured should be made in the 1.15 Notes box.* | |
| Distraction | Description |
| None | No obscuration |
| Clothes | Hoodies etc. may obscure parts of the face and neck |
| Glasses | Glasses and sunglasses may obscure eyes, completely or partially |
| Jewelry | Large jewelry may partially obscure ears and neck |
| Hat | Headgear may completely or partially cover the upper half of the face, including forehead and ears |
| Scarf | Scarves and balaclavas may cover the upper and lower half of the face, including mouth, nose and chin |
| Hair | Some hairstyles, such as bangs/fringe or long hair, may cover forehead, eyebrows, and cheeks  Facial hair may cover the lower half of the face |
| Others | Other elements, such as surgical masks, ceremonial veils may partially or totally cover facial features |

| 1.13 Expression | |
| --- | --- |
| Facial expression can affect the appearance of certain facial components.  Select using the drop-down list. | |
| Expression | Description |
| Smile (no teeth) | The lateral corners of the mouth are upturned, and the lips are closed. The lateral corners of the eyes may show crow’s feet. The teeth are not visible. |
| Smile (teeth) | The lateral corners of the mouth are upturned, and the lips are open. The lateral corners of the eyes may show crow’s feet. The upper and/or lower incisor teeth are visible and the upper and/or lower canine teeth may be visible. |
| Neutral (no teeth) | The lips are relaxed and closed with horizontal or downturned mouth corners. The eyes are open. The teeth are not visible. |
| Neutral (teeth) | The lips are relaxed and slightly open with horizontal mouth corners. The eyes are open. The incisor teeth may be partially visible. |
| (Other) | The corners of the mouth may be unevenly positioned, and the mouth is closed or open. Teeth may be visible. The eyes and nose may be distorted by the facial expression. |

| 1.14 Quality Score | |  | |  |
| --- | --- | --- | --- | --- |
| The quality score is the six-point (1 to 6) evaluation scale introduced by Schüler and Obertová (2020). Select from the drop-down list. Once the appropriate level is selected, the box will automatically turn green, yellow or red. The colours indicate the suitability of the image for comparison (see figure 7). | | | | |
| Quality Score | Description | Colour | Suitability | |
| 1  Very Good | The quality of the image corresponds well to the requirements of a morphological analysis. The resolution, sharpness, and illumination of the image are excellent. No obscuration or image artifacts. Small-scale facial features and skin structures are visible and can be described in detail. | Green | Acceptable image. Strong level of support in the evaluation phase possible. | |
| 2  Good | The quality of the image meets the requirements of a morphological analysis. Resolution, sharpness, and illumination are good. The percentage of object overlay, and image artifacts is a maximum of 5%. The small-scale facial features and skin structures are visible, but details may be unclear. |  |  |  |
| 3  Satisfactory | The quality of the image corresponds to the general requirements of a morphological analysis. A few deficits can be detected in the resolution, sharpness, or illumination. The percentage of object overlay or image artifacts is greater than 5%, but smaller than 25%. Small-scale features can be described; skin structures are not visible. |  |  |  |
| 4  Sufficient | The quality of the image is sufficient to meet the requirements of a morphological analysis. In several areas of the image, deficits are detected in the resolution, sharpness, or illumination. The percentage of object overlay and image artifacts is greater than 25%, but smaller than 75%. Small-scale features can be described to a very limited extent or not at all. | Yellow | Lower quality and restricted feature visibility. If image is used alone, a strong level of support in the evaluation phase is discouraged. | |
| 5  Poor | The quality of the image barely meets the requirements for a morphological evaluation with clear deficiencies in resolution, sharpness, and illumination. The percentage of object overlay and image artifacts is more than 75%, but less than 80%. Only large-scale features can be described. |  |  |  |
| 6  Insufficient | The quality of the image is completely insufficient for a morphological analysis. The resolution, sharpness, and illumination are deficient, or the color depth of the image is insufficient (less than 8 bit). The percentage of object overlay and image artifacts exceeds 80%. The number of visible features is insufficient for an evaluation. | Red | Very low quality, insufficient for evaluation. If only image available, level of support during the evaluation phase should be cautious. | |

**Figure 8.** Examples of the descending six-level scale of AM image quality. Each image is associated with a specific number that corresponds to the relative quality score as detailed in section 1.14 (Schüler and Obertová, 2020).

**
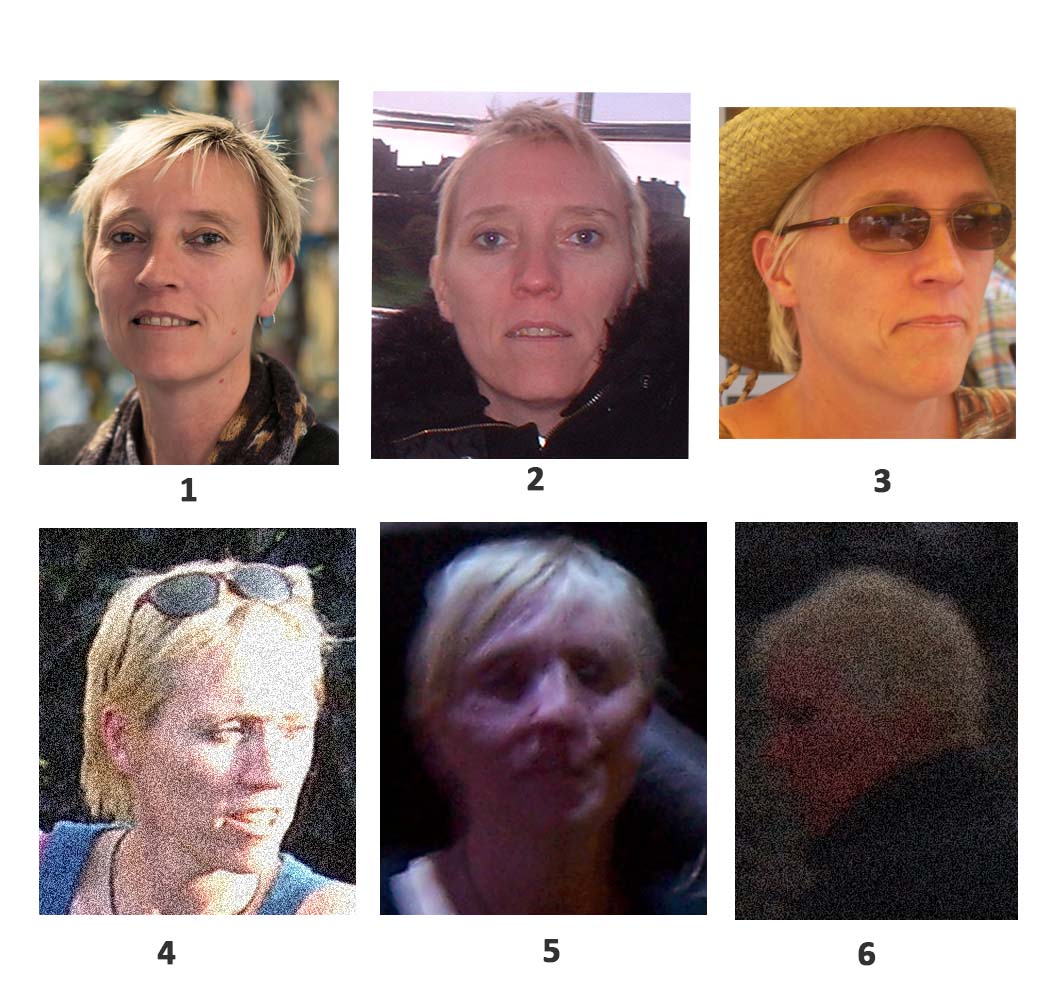
**

| 1.15 **Notes** |
| --- |
| You may add any additional information relevant to the ante-mortem analysis in this section.  This could include notes about obstructing elements. If there is no additional information to include, this section may be left empty. |

# Phase 1 – PM Analysis

Access the PM-to-AM Facial Image Comparison Excel spreadsheet and navigate to the **Phase 1-PM Analysis** tab**.** Once there, follow the below instructions to complete the columns.

During this stage, you will evaluate the suitability and quality of the PM images by assessing various image factors, decomposition changes and trauma. Notably, post-mortem changes can significantly impact the accuracy and confidence level of the final result.

Unlike AM images, which come in various formats from different sources, PM images are usually taken during post-mortem examinations with high-quality cameras using different views.

Additionally, to rank the extent of decomposition visible in each image, the protocol uses the decomposition categories and stages for the head and neck developed by Megyesi, Nawrocki and Haskell (2005).

| 1.16 PM case |
| --- |
| Insert the ante-mortem case identifier code. |

| 1.17 Total number of images |
| --- |
| Insert total number of images available for the PM case. |

| 1.18 Dental images |
| --- |
| Insert number of close-up images of dentition. |

| 1.19 Location | |
| --- | --- |
| Select the scenario where the PM images where taken. | |
| Scenario | Description |
| Scene | Images were captured at the location where the body was found. The individual may be clothed. |
| Mortuary | Images were taken during the routine autopsy. The images may depict the individual before or after the post-mortem procedure. |
| Other | Images were taken during other procedures, such as during embalming or when the deceased is in the casket. |
| Multiple | Images were taken in at least two different locations. For example, images might depict the individual where the body was found and during the autopsy. |

| 1.20 Facial Marks |
| --- |
| Any type of permanent or transient skin mark, including spots, moles, birthmarks, blemishes and freckles.  Insert a description of any observed facial marks.  ⚠ *In some cases image artifacts (due to lighting, quality etc…) might be mistaken for facial marks. State it in the description when you unsure about the nature of the mark observed.* |

| 1.21 Facial Alterations |
| --- |
| Any type of permanent skin modification, including scars, tattoos (tattoed eyebrows and lips are included) and piercings.  Insert a description of any observed alteration.  ⚠ *In some cases image artifacts (due to lighting, quality etc…) might be mistaken for scars. State it in the description when you unsure about the nature of the mark observed.*  ⚠ *In some cases individuals might be wearing fake piercings in a way that mimic the appearance of real piercings.* |

| 1.22 Decomposition Changes (from Megysei, Nawrocki, Haskell 2005) | | | |
| --- | --- | --- | --- |
| This stage employs decomposition stages for the head and neck.  The decomposition changes are classified into four categories and each category is further divided into progressive stages, which are identifiable by an abbreviation. You are required to choose the most suitable stage based on its corresponding abbreviation.  ⚠ *You do not need to manually input the decomposition score at this stage. The Decomposition Score will be calculated automatically in section 1.26.* | | | |
| Decomposition changes | | **Decomposition Score**  **(See 1.26)** | |
| 1. Fresh | 1. Fresh; no discoloration | A1 | **1pt** |
| 1. Early Decomposition | 1. Pink-white appearance with skin slippage and some hair loss | B1 | **2pts** |
|  | 1. Gray to green discoloration: some flesh still relatively fresh | B2 | **3 pts** |
|  | 1. Discoloration and/or brownish colour particularly externally; drying of nose, ears, and lips | B3 | **4 pts** |
|  | 1. Purging of decomposition fluids at eyes, ears, nose, mouth; some bloating of neck and face might be present | B4 | **5 pts** |
|  | 1. Brown to black discoloration of flesh | B5 | **6 pts** |
| 1. Advanced Decomposition | 1. Collapse of flesh and tissues of eyes and throat | C1 | **7 pts** |
|  | 1. Moist decomposition with bone exposure less than one half of the area being scored | C2 | **8 pts** |
|  | 1. Mummification with bone exposure less than one half of the area being scored | C3 | **9 pts** |
| 1. Skeletonisation | 1. Bone exposure of more than half of the area being scored with greasy substances and decomposed tissue | D1 | **10 pts** |
|  | 1. Bone exposure of more than half the area being scored with desiccated or mummified tissue | D2 | **11 pts** |
|  | 1. Bones largely dry but retaining some grease | D3 | **12 pts** |
|  | 1. Dry bone | D4 | **13 pts** |

| 1.23 Bloating |
| --- |
| Bloating is a post-mortem change that typically occurs within a few hours to several days after death. This change is a result of bacterial activity within the body, which produces gases that cause the body to swell and become distended. The face is particularly susceptible to bloating, with the most noticeable swelling occurring in the cheeks, eyelids, eyeballs, and lips. Bloating can make the face appear larger and distorted and, along with insect activity, is considered the most influential factor for changes in facial appearance (Wilkinson and Tillotson, 2012).  Describe the degree and distribution of bloating present on the face of the deceased individual. Additionally, please indicate which facial features are affected by the bloating. |

| 1.24 Trauma | |
| --- | --- |
| Severe and extensive head trauma can prevent morphological comparison. Facial trauma may include incisions, stab wounds, chops, scratches, abrasions, bruises, or bites. Select the appropriate term from the dropdown list to describe the extent of any observed trauma.  ⚠ *Provide a detailed description of injuries observed on the face in the Notes column 1.27.* | |
| Extent of facial trauma | Description |
| More than half of face | "More than half of the face" generally means that the damage or alteration to the face affects more than 50% of the total facial area. |
| Less than half of face | "Less than half of the face" typically means that the damage or alteration to the face affects less than 50% of the total facial area. |
| None | "None " means that there are no signs or indications of physical injury to the body. |

| 1.25 Obscuration | |
| --- | --- |
| Any element that obstructs or obscures facial features, including blood or other purging fluids, debris, soil, hair or clothing. Select the appropriate term from the dropdown list.  ⚠ *Provide a detailed description of any obstructed facial features in the Notes column 1.27* | |
| Extent of the facial obscuring | Description |
| More than half of face | "More than half of the face" generally means that the elements obscuring the face cover more than 50% of the total facial area. |
| Less than half of face | "Less than half of the face" typically means that the obscuring the face cover more than 50% of the total facial area. |
| None | "None " means that there are no elements obscuring the facial features. |

| 1.26 Decomposition Score (from Megysei, Nawrocki, Haskell 2005) | |
| --- | --- |
| Every decomposition stage selected in section 1.22 is associated with a corresponding numerical value 1 to 13.  Once a selection is made in column 1.22, the value will be calculated automatically. The lowest possible score a case can receive is 1, which corresponds to stage A1 (fresh, no discoloration), while the highest possible score is 13, corresponding to stage D4 (dry bone). | |
| Decomposition changes  (See 1.22) | Decomposition Score |
|  |  |
| A1 | 1pt |
| B2 | 3 pts |
| B3 | 4 pts |
| B4 | 5 pts |
| B5 | 6 pts |
| C1 | 7 pts |
| C2 | 8 pts |
| C3 | 9 pts |
| D1 | 10 pts |
| D2 | 11 pts |
| D3 | 12 pts |
| D4 | 13 pts |

| 1.27 **Notes** |
| --- |
| You may add any additional information relevant to the post-mortem analysis in this section. This could include notes about previous observations, such as traumatic injuries or obstructing elements. If there is no additional information to include, this section may be left blank. |

# Phase 2 – Facial review

Access the PM-to-AM Facial Image Comparison Excel spreadsheet and navigate to the **Phase 2-Facial Review** tab**.** Once there, follow the below instructions to complete the columns.

Facial review includes the comparison of face images holistically, recognising factors including gender, age range and skin tone, without focusing on single parts in isolation (ENFSI, 2018).

The AM and PM images are compared rapidly to determine whether two individuals are clearly not a match, prior to the detailed feature-by-feature comparison phase, by using the decision-making flowchart available below. The flowchart will then inform the decision.

| 2.1 PM case |
| --- |
| Insert the post-mortem case identifier code you are comparing. |

| 2.2 AM case |
| --- |
| Insert the ante-mortem case identifier code you are comparing. |

| 2.3 Facial review flow result | |
| --- | --- |
| Provide the correct response based on the results of the post-mortem facial comparison flow chart using the given abbreviation. | |
| Abbreviation | Description |
| P | Potential match |
| X | Exclusion |

| 2.4 Action | |
| --- | --- |
| According to the results inserted in 2.3, the action is automatically generated in this box.  No selection or insertion is required on your part. | |
| Action | Description |
| Progress to comparison | If P is entered in the 2.3 Facial Review flow results, the box will change its color to green and show the message "Progress to comparison" |
| Exclusion | If X is entered in the 2.3 Facial Review flow results, the box will change its color to red and show the message "Exclusion" |

| 2.5 **Notes** |
| --- |
| The purpose of this section is to briefly report differences found during the quick facial review and provide a rationale for why this has led to an exclusion.  Clear differences should only be used for this review and any uncertainty should lead to progression to phase 3. |

# Phase 3- Comparison and Phase 4-Evaluation

Access the "PM Photographic Facial Comparison " Excel spreadsheet and navigate to the **Phase 3-Comparison** tab**.** Once there, follow the below instructions to complete the columns.

Also, Open similar views for ante-mortem and post-mortem images. You can use any image software.

In the comparison phase, a detailed analysis of facial components, characteristics, and descriptors in both AM and PM images is conducted. To aid observations, images may be resized or rotated, but it is preferable to compare images that were captured under similar camera angles, poses, expressions, and lighting conditions (FISWG, 2018; FISWG, 2019a). During the assessment of similarities and differences, practitioners may use mark-ups and annotations such as circles, boxes, arrows, and numbers, but they should always refer back to the original images to evaluate minutiae (FISWG, 2019b). These markups and annotations should follow a consistent format in accordance with the Facial Image Comparison Best Practices for Markups and Annotations - FISWG (2019b).

**Evaluation**

Once the features have been analysed and compared, a decision must be made regarding whether each feature is similar, different, or indeterminate. Use the level of support listed in 3.16.

| 3.1 PM case |
| --- |
| Insert the post-mortem case identifier code. |

| 3.2 AM case |
| --- |
| Insert the ante-mortem case identifier code. |

| 3.3 to 3. 15 Facial Features |
| --- |
| Examine the facial features according to the instructions provided in "Facial Image Comparison Feature List for Morphological Analysis" guideline. To ensure consistency and clarity in the analysis, it is recommended to use state if the features are similar, somewhat similar, different or inconclusive.   - 3.3 Facial Marks - 3.4 Facial Alterations - 3.5 Face shape/outline - 3.6 Hairline/Forehead - 3.7 Eyebrows - 3.8 Nose - 3.9 Ears - 3.10 Mouth - 3.11 Chin and Jawline - 3.12 Facial Hair - 3.13 Facial Lines |

| 3.14 Difficult Comparison | |
| --- | --- |
| The reasons why certain comparisons may be challenging or complicated.  Select from the drop-down list. | |
| Rationale | Description |
| AM image quality | The majority of the AM images are low quality (with low resolution, poor lighting, blurriness, digital alterations). Consequently, facial details cannot easily be discerned. |
| PM changes/trauma | The extent of post-mortem changes or traumatic injuries makes photographic comparisons challenging. |
| Time lapse | AM and PM images were taken at different times/age and comparisons is difficult due to changes in appearance, such as changes in weight, hair, or skin tone. |
| Other | Other elements made the comparison difficult. |
| None | The comparison was carried out without encountering any difficulties. |

| 3.15 **Level of support** | |
| --- | --- |
| The degree of confidence that can be attributed to a particular conclusion based on the comparison of images. It is a measure of how strongly the available evidence supports a particular interpretation or conclusion.  To determine the level of support, refer to the description below and follow the instructions provided in figure 5. Choose the option from the drop-down menu. | |
| Level of support | Description |
| Strong support | The evidence provides strong support for the assertion that the images depict the same individual. |
| Moderate support | The evidence provides moderate support for the assertion that the images depict the same individual. |
| Inconclusive | The evidence is inconclusive in relation to the assertion that the images depict the same individual. |
| No support | The evidence provides no support for the assertion that the images depict the same individual. |

| 3.16 **Identifying indicators** |
| --- |
| If you choose "strong support" in column 3.16 Level of support, specify in this section the features among ears, facial marks, facial alterations (e.g., scars or tattoos) and teeth that were visible and matched between the two individuals being compared. |

| 3.17 **Notes** |
| --- |
| Can be utilised to include supplementary comments about the comparison case.  This section can be used to specify the major dissimilarities observed between the two individuals. This information can be helpful in understanding any exclusion and assist in the validation stage. |

# Phase 4- Evaluation (Summary)

Access the "PM Photographic Facial Comparison" Excel spreadsheet and navigate to the **Phase 4-Evaluation** tab**.** Once there, follow the below instructions to complete the columns.

Phase 4-Evaluation tab is interlinked with Phase 3-Comparison, displaying AM and PM analysis (3.1 and 3.2), alongside the chosen Level of Support (3.16) and the Identifying Features (3.17) determining the level of support.

| AM case |
| --- |
| Interlinked to Phase 3-Comparison Tab column 3.1 |

| PM case |
| --- |
| Interlinked to Phase 3-Comparison Tab column 3.2 |

| **Level of support** |
| --- |
| Interlinked to Phase 3-Comparison Tab column 3.15 |

| **Identifying features** |
| --- |
| Interlinked to Phase 3-Comparison Tab column 3.16 |

| **Notes** |
| --- |
| The note section can be use to include supplementary comments. |

# Stage 5- Verification

A verification stage should be employed, using an independent peer reviewer. This verification stage reduces the potential for errors and increases the reliability.
